# Predicting cognitive decline: Deep-learning reveals subtle brain changes in pre-MCI stage

**DOI:** 10.1016/j.tjpad.2025.100079

**Published:** 2025-02-06

**Authors:** Ling Yue, Yongsheng Pan, Wei Li, Junyan Mao, Bo Hong, Zhen Gu, Mingxia Liu, Dinggang Shen, Shifu Xiao

**Affiliations:** a Department of Geriatric Psychiatry, Shanghai Mental Health Center, Shanghai Jiao Tong University School of Medicine, 600 South Wanping Road, 200032, Shanghai, China; b Alzheimer's Disease and Related Disorders Center, Shanghai Jiao Tong University, 600 South Wanping Road, 200032, Shanghai, China; c School of Computer Science and Engineering, Northwestern Polytechnical University, 127 West Youyi Road, 710072, Xi'an, China; d Department of Radiology and BRIC, University of North Carolina at Chapel Hill, 130 Mason Farm Road, Chapel Hill, NC 27599, USA; e School of Biomedical Engineering & State Key Laboratory of Advanced Medical Materials and Devices, ShanghaiTech University, Shanghai 201210, China; f Shanghai United Imaging Intelligence Co., Ltd., Shanghai 200232, China; g Shanghai Clinical Research and Trial Center, Shanghai, 201210, China

**Keywords:** Deep-learning, Region-of-interest, Mild cognitive impairment, Prediction, MRI

## Abstract

**Background:**

Mild cognitive impairment (MCI) and preclinical MCI (e.g., subjective cognitive decline, SCD) are considered risk states of dementia, such as Alzheimer's Disease (AD). However, it is challenging to accurately predict conversion from normal cognition (NC) to MCI, which is important for early detection and intervention. Since neuropathological changes may have occurred in the brain many years before clinical AD, we sought to detect the subtle brain changes in the pre-MCI stage using a deep-learning method based on structural Magnetic Resonance Imaging (MRI).

**Objectives:**

To discover early structural neuroimaging changes that differentiate between stable and progressive cognitive status, and to establish a predictive model for MCI conversion.

**Design, setting and participants:**

We first created a unique deep-learning framework for pre-AD conversion prediction through the Alzheimer's Disease Neuroimaging Initiative-1 (ADNI-1) database (*n* = 845). Then, we tested the model on ADNI-2 (*n* = 321, followed 3 years) and our private study (*n* = 109), the China Longitudinal Aging Study (CLAS), to validate the rationality for pre-MCI conversion prediction. The CLAS is a 7-year community-based cohort study in Shanghai. Our framework consisted of two steps: 1) a single-ROI-based network (SRNet) for identifying informative regions in the brain, and 2) a multi-ROI-based network (MRNet) for pre-AD conversion prediction. We then utilized these "ROI-based deep learning" neural networks to create a composite score using advanced algorithm-building. We coined this score as the Progressive Index (PI), which serves as a metric for assessing the propensity of AD conversion. Ultimately, we employed the PI to gauge its predictive capability for MCI conversion in both ADNI-2 and CLAS datasets.

**Measurements:**

We primarily utilized baseline T1-weighted MRI scans to identify the most discriminative brain regions and subsequently developed the PI in both training and validation datasets. We compared the PI across different cognitive groups and conducted logistic regression models along with their AUCs, adjusting for education level, gender, neuropsychological test scores, and the presence of comorbid conditions.

**Results:**

We trained the SRNet and MRNet using 845 subjects from ADNI-1 with baseline MRI data, in which AD and progressive MCI (converting to AD within 3 years) patients were considered as positive samples, while NC and stable MCI (remaining stable for 3 years) subjects were considered as negative samples. The convolutional neural networks identified the top 10 regions of interest (ROIs) for distinguishing progressive from stable cases. These key brain regions included the hippocampus, amygdala, temporal lobe, insula, and anterior cerebellum. A total of 321 subjects from ADNI-2, including 209 NC (18 progressive NC (pNC), 113 stable NC (sNC), and 78 remaining NC (rNC)) and 112 SCD (11 pSCD, 5 sSCD, and 96 rSCD), as well as 109 subjects from CLAS, including 17 sNC, 16 pNC, 52 sSCD and 24 pSCD participated in the test set, separately. We found that the PI score effectively sorted all subjects by their stages (stable vs progressive). Furthermore, the PI score demonstrated excellent discrimination between the two outcomes in the CLAS data(p<0.001), even after controlling for age, gender, education level, depression symptoms, anxiety symptoms, somatic diseases, and baseline MoCA score. Better performance for prediction progression to MCI in CLAS was obtained when the PI score was combined with clinical measures (AUC=0.812; 95 %CI: 0.725–0.900).

**Conclusions:**

This study effectively predicted the progression to MCI among order individuals at normal cognition state by deep learning algorithm with MRI scans. Exploring the key brain alterations during the very early stages, specifically the transition from NC to MCI, based on deep learning methods holds significant potential for further research and contributes to a deeper understanding of disease mechanisms.

**Key points**

**Questions:** What is the distinctive performance of early, subtle changes in structural MRI during pre-mild cognitive impairment (pre-MCI) stage?

**Findings:** In our deep-learning model, we used the ADNI-1 dataset (with 845 subjects) for training and the ADNI-2 (with 321 subjects) and CLAS (with 109 subjects) datasets for validation. The model was designed to identify a progressive index (PI), underscoring the importance of medial temporal and cerebellum structures in its good classification ability.

**Meaning:** This study developed the PI to synthetically assess the prognosis from normal cognition to MCI, aiming to facilitate early detection of cognitive deterioration.

Alt-text: Unlabelled box

## 1 Background

Mild cognitive impairment (MCI) is a condition in which an individual experiences a decline in cognitive function that is noticeable to themselves or others, but does not significantly interfere with daily activities or independence [1]. MCI is considered as a transitional stage between healthy aging and dementia, affecting 10–15 % of the population over the age of 65 [2]. The progression from MCI to any form of dementia is 3 to 5 times more likely than people with normal cognition, with annual progression rates of 12 % in the general population and up to 20 % in high-risk individuals [3]. It's worth noting that, not all individuals with MCI will inevitably turn into dementia. With early recognition and intervention, a significant proportion of individuals with MCI can return to normal or maintain their current cognitive status [4]. Therefore, identifying individuals at the preclinical MCI (pre-MCI), is fundamental for early intervention of pathologic cognitive decline [5].

Currently, two main groups of biomarkers can assist in the diagnosis of MCI: amyloid beta (Aβ) deposits (such as cerebrospinal fluid concentrations of Aβ42 and positron emission tomography (PET) amyloid imaging) and neuronal damage (such as CSF tau/phosphorylated tau, hippocampal volume or medial temporal atrophy, rate of brain atrophy, fluorodeoxyglucose (FDG) PET imaging and SPECT perfusion imaging) [6]. Although the technologies mentioned above show high categorical accuracy, these methods are generally not available in primary clinical settings, given the cost and invasiveness of the procedure. In clinical practice, general demographic data, neuropsychological tests, and MRI are often available. There is an urgent need to use these multimodal data to predict whether an individual will develop MCI or dementia in the future.

Deep-learning frameworks are software libraries that allow developers to create and train learning-based models. These frameworks can provide a wide range of tools and functionalities to help developers build complex deep neural networks and train them on large datasets [7,8]. Previous studies have shown that deep-learning frameworks can be used to analyze large-scale medical data, such as brain scans or cognitive test results, to identify patterns and relationships that may be indicative of MCI. Moreover, these algorithms can also be used to predict which individuals are at greater risk of developing MCI [9,10] or progressing from MCI to dementia [[11], [12], [13]].

As there are relatively few similar studies in China and the follow-up time is also relatively short, to fill in the existing research gaps, two large databases of Alzheimer's Disease Neuroimaging Initiative (ADNI) and China Longitudinal Aging Study (CLAS) were used in this work. Our study aimed to explore whether structural MRI data can be used to predict the future risk of MCI in normal older adults by using artificial intelligence (AI) technology.

## 2 Methods

### 2.1 Cohort study information

The dataset consists of two databases, the ADNI database and the CLAS database. The Alzheimer's Disease Neuroimaging Initiative (ADNI) database is a large-scale study launched in 2004 to improve the understanding, diagnosis, and treatment of Alzheimer's disease (AD). It collects and analyzes data from hundreds of participants, including individuals with Alzheimer's disease, mild cognitive impairment, and healthy controls. The data includes clinical, neuropsychological, imaging, genetic, and biomarker information, which is used to track the progression of the disease and to identify potential new treatments [14].

The China Longitudinal Aging Study (CLAS) is a large-scale longitudinal study that examines the health and well-being of older adults in China initiated in 2012 [15]. The main objective of the study is to understand the dynamics of aging and its impact on the health and quality of life of older adults in China. CLAS collects data on a wide range of factors that affect aging, including demographic information, health status, lifestyle habits, social support, economic status, and healthcare utilization. The study has a nationally representative sample of older adults aged 60 and above, and it includes both urban and rural areas of China [15]. The current data constitute samples of individuals from Shanghai, defined as NC, who received a T1-weighted MRI scan at baseline and finished the 7-year follow-up. Unfortunately, the elderly participants in CLAS cohort were not followed up during the seven-year period.

### 2.2 Research process

**Fig. 1:**
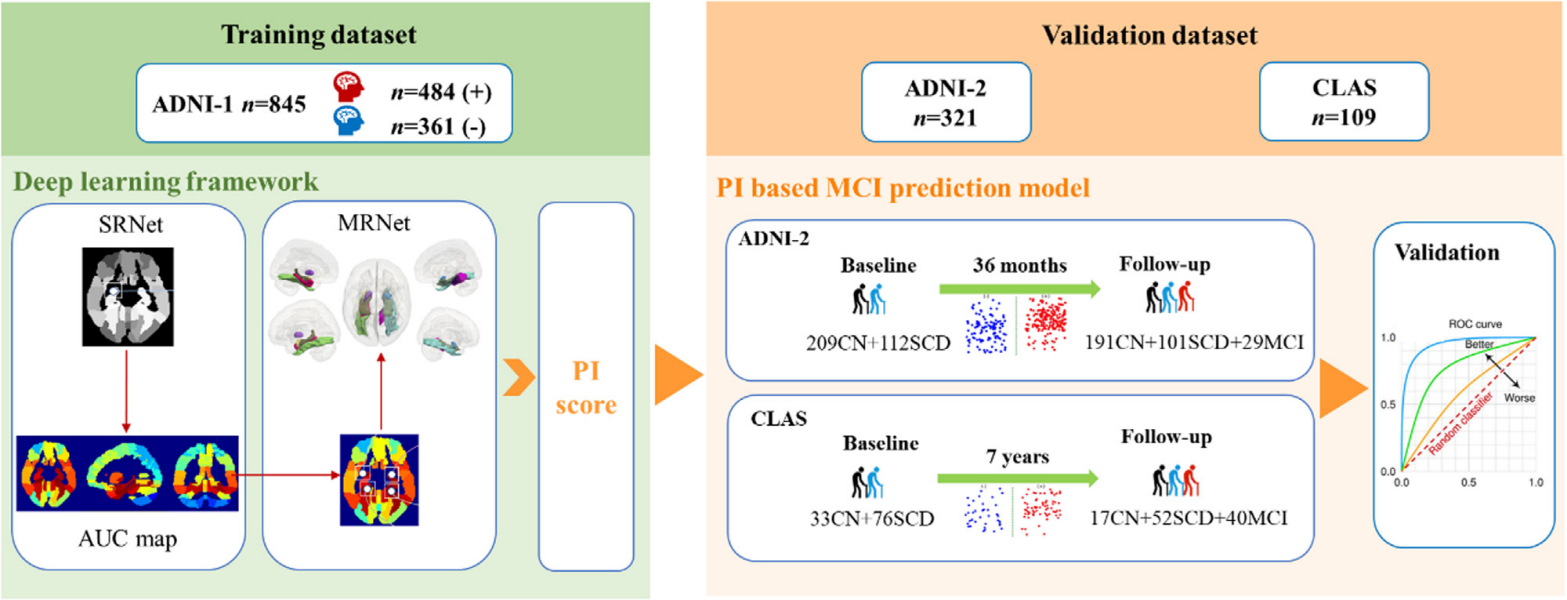
Flowchart of the primary study. Note: NC, cognitively normal; SCD, subjective cognitive decline; MCI, mild cognitive impairment; SRNet, single-ROI-based network; MRNet, multi-ROI-based network; PI, progressive index.

The overall procedure is delineated in Fig. 1. The baseline neuroimaging data consisted of T1-weighted MRIs from 845 ADNI-1, 321 ADNI-2, and 109 CLAS subjects. We used ADNI-1 MRI data (*n* = 845) to train the proposed deep-learning model. We then validated the proposed model on independent data from ADNI-2 and the CLAS. Both ADNI-1 and ADNI-2 cohort subjects had a 36-month follow-up. For independent training and testing, participants who were involved in both ADNI-1 and ADNI-2 are excluded from ADNI-2. Meanwhile, 109 CLAS subjects with normal cognitive function also underwent head MRI scans at baseline and were clinically followed up after seven years. At follow-up, 40 subjects converted to MCI, while 69 subjects remained cognitively normal. The characteristics of the CLAS featured in this analysis are detailed in Table 1. Additionally, eTable1 and eTable2 provide overviews of ADNI-1 and ADNI-2 included in this study, respectively. (see **Supplementary Materials**).

**Table 1:** Demographics for the CLAS dataset.

	sNC (*n* = 69)	pNC (*n* = 40)	t/λ^2^
**Baseline age**	66.72(5.59)	70.88(6.31)	−3.561**
**Years of education**	9.88(2.98)	7.18(4.28)	3.534**
**Gender (Male/female)**	30/39	19/21	ns.
**Follow-up interval, months**	83.82(1.03)	83.92(1.18)	ns.
**Smoke, year**	10.88 (16.90)	7.60 (16.28)	ns.
**Drink, year**	5.33 (11.90)	6.08 (14.29)	ns.
**History of stroke (Yes/no)**	2/67	6/34	0.020*
**Surgery history (Yes/no)**	34/32	14/26	ns.
**Heart disease(yes/no)**	15/54	10/30	ns.
**Hypertension, year**	6.17 (9.18)	5.99 (8.62)	ns.
**Hyperlipidemia, year**	1.36 (4.69)	1.18 (4.27)	ns.
**Diabetes mellitus, year**	1.66 (6.78)	1.38 (5.03)	ns.
**GDS**	2.94 (4.228)	1.68 (2.464)	ns.
**SAS**	23.73 (3.473)	22.39 (3.115)	ns.
**Social support score**	36.35 (9.575)	33.35 (9.937)	ns.
**MMSE**	28.12(1.94)	27.15(2.37)	0.032
**MoCA**	25.10(3.70)	23.60(4.40)	ns.
**Digit_span_forward**	9.54 (2.41)	9.07 (2.44)	ns.
**Digit_span_backward**	5.93 (2.40)	5.35 (2.05)	ns.
**AVLT_TOTAL**	33.7 (8.99)	31.2 (10.2)	ns.
**Associative_learning**	7.41 (3.40)	5.18 (2.77)	3.730**
**Visual_recognition_functional**	3.61 (0.65)	3.38 (0.81)	ns.
**Visual_recognition_semantic**	3.19 (0.94)	3.15 (1.08)	ns.
**Visual_matching_reasoning**	6.03 (2.29)	5.20 (2.36)	ns.
**Visual_recognition_correct**	6.26 (1.29)	5.92 (1.42)	ns.
**Verbal_fluency**	9.74 (3.17)	8.83 (3.34)	ns.
**WAIS_picture_completion**	11.4 (4.24)	9.97 (4.02)	ns.
**WAIS_block_design**	29.3 (7.51)	26.7 (7.84)	ns.
**Progressive Index (PI)**	0.31(0.13)	0.42(0.15)	16.298**

Note: p, progressive; s, stable; * and ** denotes *p* < 0.05 and *p* < 0.01, respectively; GDS: Geriatric Depression Scale; SAS: Self-rating Anxiety Scale.

### 2.3 Deep-learning framework for pre-AD prediction

**Fig. 2:**
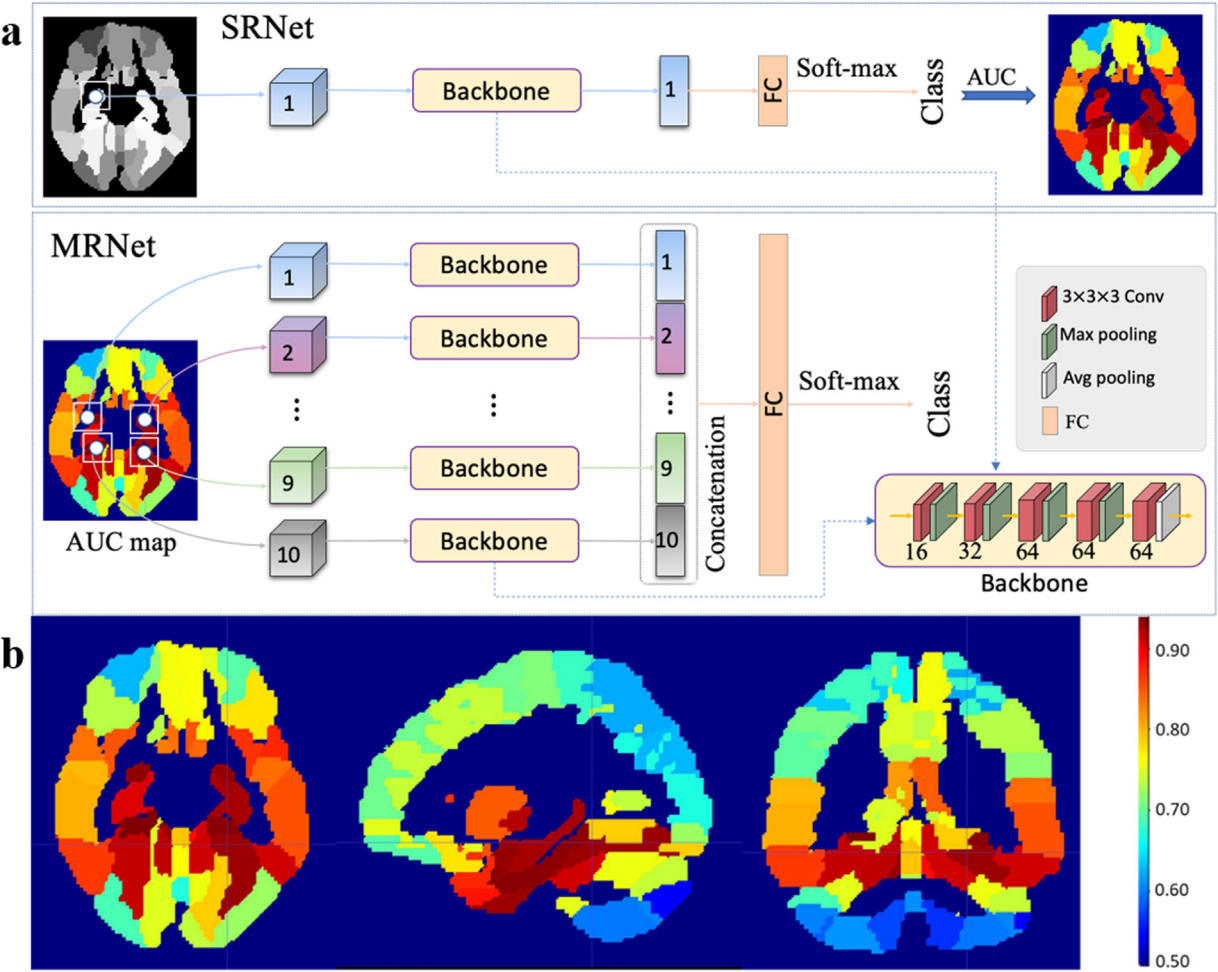
*a) Overview of SRNet and MRNet.* Structures of our SRNet (top) with 1 backbone and 1 FC layer, and our MRNet (bottom) with 10 parallel backbones and 1 FC layer. Each backbone contains five 3 × 3 × 3 convolutional layers with ReLU activation and max pooling for the first 4 layers and average pooling for the last layer. *b) AUC values achieved by SRNet in AD* vs*. NC classification for each ROI.* The top 10 ROIs are selected as the discriminative regions in the brain. Here, red denotes a high AUC value, while blue denotes a low AUC value.

Motivated by previous disease diagnosis systems with MRI [16], we created a unique deep-learning framework for pre-clinical AD (pre-AD) conversion prediction by evaluating the discriminative capability of single region-of-interest (ROI) or multiple ROIs in the brain. Our framework is illustrated in Fig. 2**a**, which includes 2 steps: 1) a single-ROI-based network (SRNet) for locating informative regions in the brain, and 2) a multi-ROI-based network (MRNet) for pre-AD conversion prediction. The backbone modules in both SRNet and MRNet share the same architecture, which consists of five 3 × 3 × 3 convolutional layers with rectified linear unit (ReLU), and the channels for these convolutional layers are 16, 32, 64, 64 and 64, respectively. The former 4 convolutional layers are followed by max-pooling with a 2-voxel stride, and the 5th layer is followed by avg-pooling. The input of each backbone is a local patch (size: 32 × 32 × 32), and the output is a 64-dimensional vector.

In the 1st step, we designed the SRNet to evaluate the discriminative capability of each of the 116 ROIs in the AAL template [17]. The SRNet contained a single backbone (corresponding to an ROI) and 1 fully-connected (FC) layer followed by the soft-max activation. We first trained each SRNet for each ROI based on the patches extracted at the centroid of this ROI to record the AUC value in AD vs. NC classification. We then ranked these ROIs according to their discriminative capabilities (regarding AUCs) and select the top 10 ROIs (with the highest AUC values) as brain locations (see Fig. 2**b**).

In the 2nd step, we trained the MRNet for pre-AD conversion prediction. The MRNet first stacked 10 backbones, and then concatenated their outputs for a subsequent FC layer. These 10 parallel backbones shared the same parameters, while the length of this FC layer was ten times that in SRNet. Its input consisted of 10 local patches centered at pre-selected ROIs in each MRI, while its output was the corresponding class label for each subject.

In both the 1st and 2nd steps, we used 845 subjects with baseline MRI in ADNI1 [18] to train SRNet and MRNet. All images are processed with skull-stripping and spatial normalization. According to previous studies, some individuals with MCI remain stable after several years, with trajectories of cognition similar to those in normal aging [19]. Hence, we presume that NC and stable MCI possess similar structural characteristics, which contrast sharply with those of progressive MCI and AD. To balance the sample size in each category, we use both AD and progressive MCI (within 3 years) patients as positive samples, while NC and stable MCI (within 3 years) subjects as negative samples. The output of MRNet is the probability of each subject converting to MCI within 3 years.

### 2.4 PI formulation

We aim to construct a computer-aided diagnosis system (denoted as F) to convert a given brain image (e.g., T1-weight MRI, denoted as M) to an index $\hat{y}$ to indicate the progression of a specific brain disorder, such as Alzheimer's disease.

Traditional studies typically regarded the state of a subject as a binary class label, e.g., y=1 for positive and y=0 for negative subjects, and construct classification models that can only determine a subject is either 1/positive or 0/negative. However, such a process cannot reflect the intermediate states of a gradual degeneration progression. For example, a subject affected by AD at 70 years old (state denoted as 0, cognitively normal) and diagnosed with AD at 80 years old (state denoted as 1), previous studies can only roughly indicate the states at 71, 72, …, 79 years old to be 0 or 1 while the actual states shall be fuzzy numbers between 0 and 1.

To this end, we propose to create a continuous index (denoted as PI) that can reflect the current state (ranging from 0 to 1) of a subject based on his/her MRI scan, thus providing intuitive prompts for clinical decision-making. The most challenging aspect of this task is that we only have the binary decisions (0 or 1) of training subjects, while we want to predict the continuous indexes (ranging from 0 to 1) for test subjects. To track this, we propose to construct our system by employing a deep-learning framework that requires binary class labels as targets but outputs the continuous probability of subjects being positive, which could be used as the progressive index (PI).

In this framework, a deep-learning model (denoted as F) can be learned by minimizing the following cross-entropy loss

L=−[ylogF(M)+(1−y)log(1−F(M))],

which encouraging the output PI close to its ground-truth diagnosis label.

During inference for a new subject, we can directly input its MRI $\hat{M}$ to the framework and obtain the estimated PI as follows.

$$\hat{y}=F\left(\hat{M}\right)$$

Thus, PI is a score in the range of [0, 1] that could be used as the progressive index (PI) to measure this subject's current progress. Intuitively, a higher score/PI indicates that this subject is very likely to progress to MCI.

### 2.5 Validation for MCI prediction based on ADNI-2

We used SCD and NC subjects from an independent ADNI-2 dataset (http://adni.loni.usc.edu/), another widely adopted longitudinal dataset, for tracking pre-MCI progression to validate our model. We then applied the model trained on ADNI-1 to the independent ADNI-2 dataset (with 321 subjects, including 209 NCs and 112 SCDs). Among these 321 test subjects, 18 NC (progressive NC, pNC) and 11 SCD (progressive SCD, pSCD) subjects were absolutely converted to MCI within 3 years, 113 NC (stable NC, sNC) and 5 SCD (stable SCD, sSCD) subjects were absolutely not converted to MCI, and the remaining 78 NC (remaining NC, rNC) and 96 SCD (remaining SCD, rSCD) subjects were unsure due to lacking of the 3rd-year scans. The follow-up time for the ADNI data was 45.39±7.89 months (∼ 4 years). The PI was achieved by the similar ROI-based deep-learning method. Then the general linear model was used to compare the difference between the progressive and stable groups.

### 2.6 Validation for MCI prediction based on CLAS

Then, we applied the model trained on ADNI-1 to our private——CLAS data and obtained the intuitive PI score according to the algorithm. The CLAS data was followed for 83.86±1.09 months (∼7 years). Among 109 cognitively normal subjects (33NC and 76 SCD at baseline), 16 NC and 24 SCD showed clinical progression to amnestic MCI at follow-up. To facilitate clinical application, the general linear model was used to compare PI between the progressive and stable groups. We drew a receiver operating characteristic (ROC) curve and used the area under the curve (AUC) to measure the discriminant ability in the prediction of MCI progression.

In the first step, in order to find the candidate discriminative features, all the clinical data were assessed in a univariate analysis. After that, the regional brain variables were further analyzed using binary logistic regression in two models. We treated the group as a dependent variable, and in Model 1 using gender, age, years of education and brain size index as covariates. As previous literature confirmed that a few major factors accounting for AD include depression, hypertension, diabetes, physical inactivity, smoking [20], so in Model 2, we additionally adjusted for GDS score, self-report anxiety, hypertension, hyperlipidemia, diabetes mellitus, heart disease and smoking as covariates. Contrasts were calculated by SCD vs. NC, MCI vs. NC, and SCD vs. MCI, respectively. Pearson's correlation analysis was performed to examine relationships between structural data and neuropsychological performances using age as covariate.

## 3 Results

### 3.1 ROI-based deep-learning brain network

**Fig. 3:**
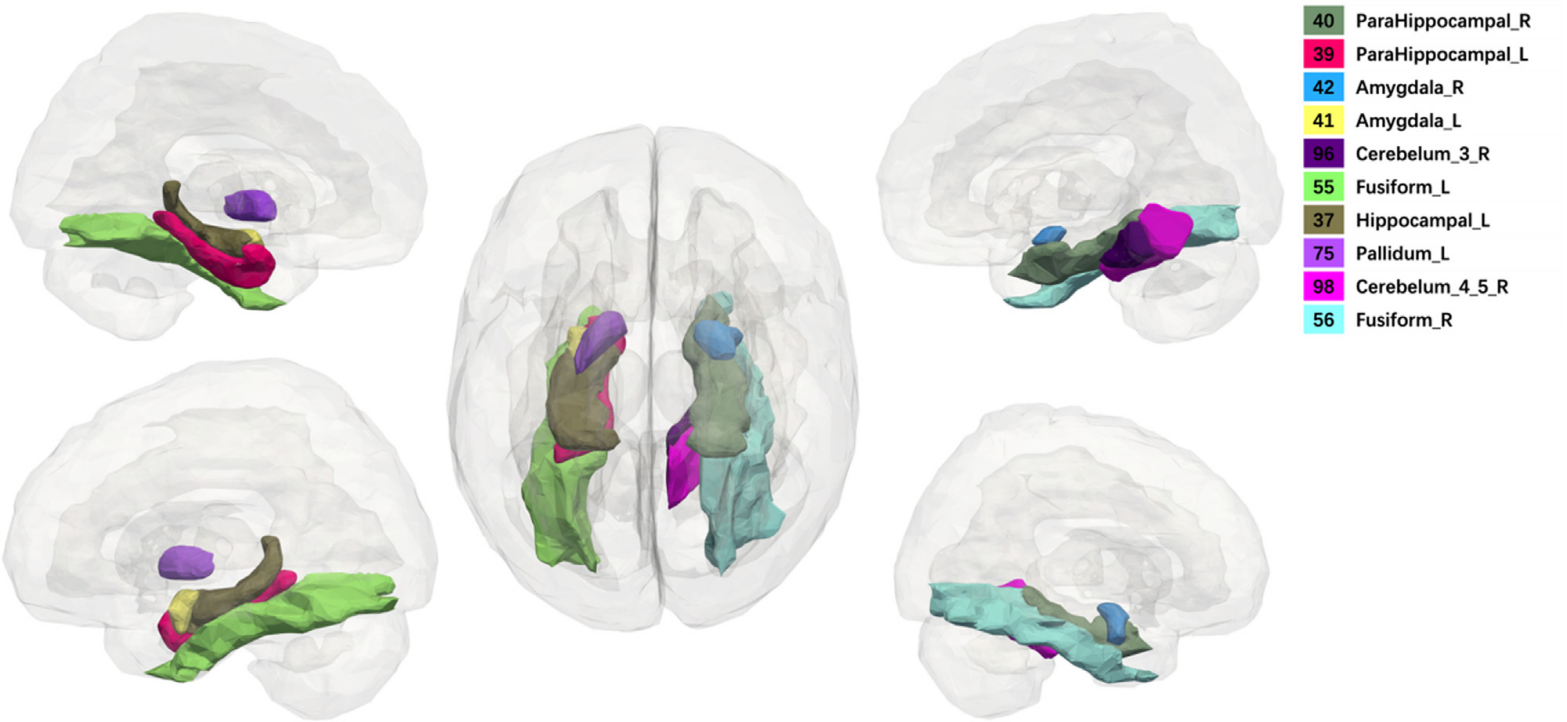
Top 10 discriminative brain regions detected by the proposed ROI-based deep-learning approach in MCI prediction. L and R denote left and right respectively. Cerebelum_3_R: Right Cerebellum_Superior (96, SRI24 code); Cerebelum_4_5_R: Right Cerebellum_Superior (98, SRI24 code).

Our predictive model identified the top 10 discriminative brain regions in AD from ADNI-1 dataset using our framework. These regions include the bilateral amygdala, bilateral parahippocampus, bilateral fusiform, left hippocampus, left pallidum, and right part of cerebellum, respectively (Fig. 3). For further details on the cerebellar regions segmented by the AAL template, please refer to the comprehensive information provided by the SRI24 template [21].

### 3.2 Performance of MCI prediction model in ADNI-2 dataset

**Fig. 4:**
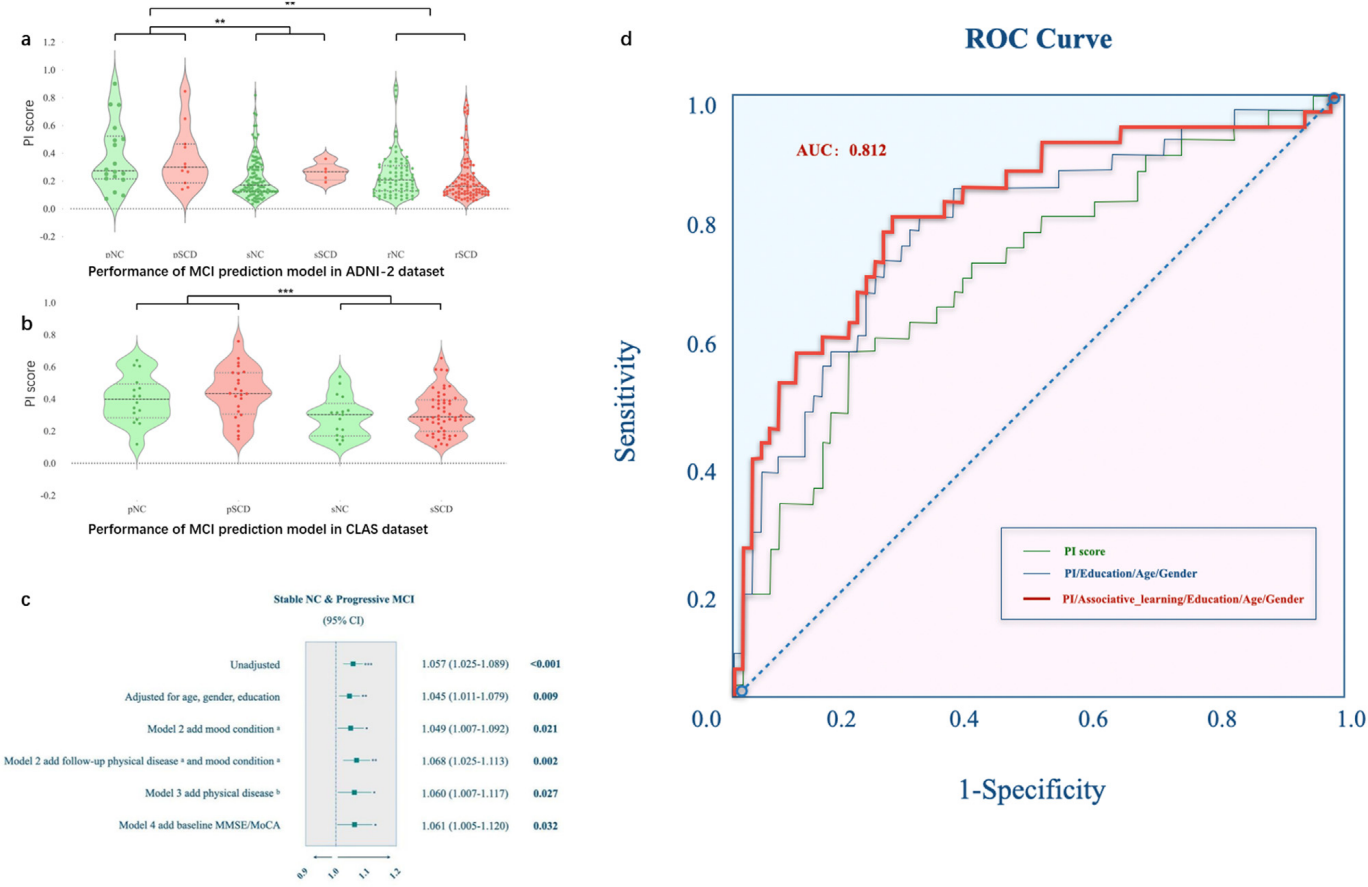
Performance of MCI prediction model in ADNI-2 and CLAS datasets. *(a) Performance of MCI Prediction model in ADNI-2 dataset.* MRNet used an ADNI-1 trained model to calculate the PI score for 209 NCs and 112 SCDs in ADNI-2. The x-axis in the graph represents subjects categorized by their cognitive stages: 18 progressive NC (pNC), 113 stable NC (sNC), and 78 remaining NC (rNC), as well as 11 pSCD, 5 sSCD, and 96 rSCD. *(b) Performance of MCI Prediction model in CLAS dataset.* MRNet also estimated the PI for 109 subjects in the CLAS study, again using the ADNI-1 trained model. The subjects are indexed and categorized by their stages: 17 sNC, 16 pNC, 52 sSCD and 24 pSCD. Stars on the graph mark the mean PI for each group. *(c) Logistic regression analysis among sNC and pNC after multivariable adjustment.*^a^Mood condition including GDS/SAS/social support score. ^b^Physical disease including baseline diabetes mellitus/hyperlipidemia/stroke/heart disease/hypertension/smoke/drink. *(*d*) ROC curves of MCI prediction models built on different feature combinations.*

Fig. 4a described that the subjects in ADNI-2 were roughly sorted by their stages by using the ROI-based framework. In ADNI-2, the overall average PI was 0.2449, while those of pNC, sNC, pSCD and sSCD groups were 0.3763, 0.2240, 0.3688 and 0.2663, respectively. The PI of pNC and pSCD were obviously higher than those of other groups. We further tested the hypothesis that pNCs and pSCDs had higher scores than others, which was supported by the resulting ***p*-value** of 0.0027. This suggests brain structure changes at the pre-MCI stage could be objectively detected in MR images by our method. The AUC value was 0.7048, and the SPE and SEN were 0.6667 and 0.6897 (with the threshold of the overall average score).

### 3.3 The performance of MCI prediction model on CLAS dataset

The demographic and clinical characteristics of the 109 NC subjects in CLAS dataset are presented in Table 1. At the 7-year follow-up after the baseline time, 17pNC developed into amnestic MCI with significantly lower MoCA and MMSE scores but still comparable physical disease statuses and the GDS score compared to sSCDs. In CLAS, the overall average PI was 0.3491, while those of sNC, pNC, sSCD and pSCD groups were 0.2881, 0.3988, 0.3144 and 0.4343, respectively (See Fig. 4**b**). The AUC value was 0.711, and the SPE and SEN were 0.575 and 0.812.

To address the possible confounding effect, logistic regression analysis was performed to assess the association between PI score and two different outcome groups in a series of models (see Fig. 4**c**). Between stable and progressive groups, significant differences were found in the PI value (odds ratios (OR) for unadjusted: 1.057, p<0.001, model 1(adjusted for age, gender and education): 1.045, *p* = 0.009, and model 2(add mood condition): 1.049, *p* = 0.021), model 2 add follow-up physical disease (OR for model 1: 1.068, *p* = 0.002, and model 4 add baseline MMSE: 1.061, *p* = 0.032).

To facilitate clinical applications, we combined demographic information, neuropsychological scale and the PI score from MRI scans to establish a model for MCI prediction. Through logistic regression analysis, we found that the PI value combined with a neuropsychological scale score (associative learning) and General demographic information (age, gender and years of education) can effectively predict the conversion of MCI in the CLAS cohort after 7 years, with an AUC of 0.812, and the SPE and SEN were 0.800 and 0.739 (see Fig. 4**d**).

## 4 Discussion

Effectively and accurately predicting MCI progression when older persons are cognitively normal is of great importance for initiating early interventions or disease-modifying treatments. In the current study, we used two distinct databases, ADNI and CLAS, for simultaneous verification. Ultimately, we proposed an innovative and clinically feasible algorithm from ten specific brain regions to achieve promising performance in predicting the progression from NC to MCI. Furthermore, we found that the combination of ROI-based deep-learning scores and clinical indicators may predict MCI conversion 7 years before onset with satisfactory accuracy (AUC=0.812). Our findings demonstrated that even in the pre-MCI stage, a deep-learning-based prediction model utilizing MRI data can predict future MCI conversion 7 years before onset.

The basic idea behind a ROI-based deep-learning framework is to incorporate relational reasoning into the learning process, which allows the model to capture complex patterns and dependencies in the data. Overall, ROI-based deep-learning frameworks represent an important and promising direction in the field of artificial intelligence, as they enable models to reason about complex relationships and dependencies in the data. This is crucial for a wide range of applications, including natural language processing, computer vision, and robotics. The introduction of ROI-based deep-learning frameworks into the field of neurocognition is a major innovation of our research. Compared with traditional statistical analysis methods, it can analyze and include more variables, so it is more likely to find more novel predictive factors.

With the development of machine learning algorithms, more and more dementia risk prediction models are being developed. At present, there have been some studies using ROI-based deep-learning frameworks to predict the occurrence and development of MCI. For example, Kam TE et al. found that ROI-based deep-learning framework could effectively use resting-state functional MRI (rs-fMRI) and the derived brain functional networks (BFNs) to predict the onset of MCI [22]. In Lee J et al.’ study, they proposed a new framework based on functional MRI to identify subjects with early mild cognitive impairment (eMCI) and account for individual differences by simultaneously learning functional relationships from each subject's automatic selection region of interest (ROI) [23]. Moreover, in Skolariki K et al.’s study, they also found that the accuracy of the ROI-based deep-learning framework in predicting the transition of MCI to dementia was up to 84 % [24]. In our study, we also found that ROI-based deep-learning framework predicted MCI with an area under ROC curve of 0.7048, which also showed high prediction efficiency. Therefore, this method has high application potential and development value.

The strength of this study lies in the development of a PI score that encapsulates the insights from deep-learning image features to assess the risk of MCI/AD conversion. Furthermore, this PI score facilitates clinicians in not only comprehending the risk level effortlessly but also in its practical application, such as its integration with baseline clinical measurements. Additionally, a previous study reported improved performance for predicting AD progression by using the deep-learning hippocampal imaging features and clinical measures (demographic information and ADAS-Cog13) [25], which is similar to our result that PI score, combined with basic information (age, gender, education), and association learning score could predict NC to MCI conversion sufficiently even 7 years before (AUC=0.812). Recently, another study reported combination of MRI and genetic features improved the time-to-event prediction for subjects with MCI, while the combination of cognitive tests, demographic, and CSF data showed better performance compared with genetic or MRI data in predicting AD conversion [26]. Hence, the use of extensive neuropsychological scales in early screening for cognitive impairment can't be ignored.

Remarkably, our study identified a combination of ten core brain regions associated with cognitive decline risk, indicating a potential for a more comprehensive and sensitive biomarker. This finding suggests that the integration of multiple brain regions, rather than a single subregion, offers a more thorough reflection of the pathophysiological changes in Alzheimer's disease (AD). A meta-analysis has highlighted the role of AI technology in predicting AD, particularly in capturing and quantifying subtle MRI changes across the brain [27]. This exemplifies the complexity and heterogeneity of AD and brain aging. Among the regions identified, the hippocampus and amygdala are most frequently reported as the initial areas affected in AD, and the extent of abnormalities in these regions may reflect disease severity [[28], [29], [30]]. Meanwhile, it is worth mention that only the left hippocampus was outputted as a discriminative feature. Our previous research [31] has identified lateralized patterns in the hippocampus among individuals of SCD and MCI. This pattern showed that the atrophy of the right hippocampus is more pronounced than that of the left during the early stages of cognitive impairment. However, a meta-analysis [32] showed a contrary pattern: a consistent left-less-than-right asymmetry pattern is found, but with different extents in control, MCI, and AD group. Furthermore, one study [33] revealed solely left hippocampus volume can particularly discriminate stable and progressive MCI. It remains unclear whether the left, right, or both hippocampi influence cognitive decline. Meanwhile, parahippocampal and fusiform are also important brain regions in early AD detection [34,35]. The parahippocampal gyrus, a diverse and intricate anatomical structure, exhibits notable clinical and preclinical neuroanatomical correlations with Alzheimer's disease (AD) [36]. A neuroimaging study found that parahippocampal volume discriminates better than hippocampal volume in the early phase of AD [37]. The fusiform gyrus, situated between the inferior temporal gyrus and parahippocampal gyrus, is also an early victim of AD. The region is involved in diverse cognitive functions like color processing, face/body recognition, intra-category identification, and word comprehension. Furthermore, it is noteworthy that our deep-learning model selectively focused on two distinct subregions of the cerebellum. The cerebellum, a vital component of the distributed neural networks, is not solely involved in motor functions but also plays a pivotal role in cognitive behaviors. Extensive research has revealed the existence of intrinsic connectivity networks that precisely align with the cerebrocerebellar connections in a topographically organized manner [38]. Moreover, sufficient evidence suggests that the cerebellum performs a compensatory function during the initial stages of Alzheimer's disease (AD), because of the observed regional hypermetabolism in the cerebellum in AD patients [39]. Our findings, utilizing the deep-learning method, validate the perspective that the cerebellum plays a pivotal role beyond a mere observer in the pathophysiology of AD. Future exploration is needed to understand the mechanisms of how AD pathology develops within the cerebellum by using AD biomarkers, such as amyloid PET. An improved understanding of different subregions of the cerebellum will aid the researchers in elucidating the mechanism of memory disorders.

We have to admit that our research has some limitations. First of all, we only included two cohorts and did not verify our data through collaboration with other research institutions. Second, we did not include classical AD biomarkers, such as APOE E4, Aβ protein and tau protein. MCI diagnosis does not mean AD pathology in the brain, but it is hard to conduct CSF or PET scans in a community-based cohort. However, our 7-year longitudinal findings still reflect the actual cognitive outcomes, and further long- term follow-up is needed in the future. Third, our study lacks annual follow-ups, which could potentially assist in promptly recognizing the transition of NC to MCI. Fourth, we have currently only used T1-weighted structural MRI. In the future, the proposed deep-learning framework has the potential for enhanced predictive performance by incorporating additional neuroimaging modalities, including T2-weighted MRI, Diffusion Tensor Imaging (DTI), and resting-state functional MRI.

This study provides a new, effective, deep-learning and MRI-based method to predict the risk of developing MCI with unprecedentedly long follow-up time among cognitively normal older individuals. Our long-term clinical follow-up study accurately assessed the effectiveness and reliability of this potential multi-regional biomarker for AD. The combination of these brain regions and clinical features has the potential to serve as a more sensitive and specific biomarker for early diagnosis and disease monitoring of AD. This method is expected to be used in clinical practice for early diagnosis and intervention to improve the quality of life of older individuals.

## Ethical standards

The study protocol was approved by the Ethical Committee of the Shanghai Mental Health Center (No. 2012–019). All human subjects provided informed consent.

## Declaration of generative AI and AI-assisted technologies in the writing process

We did not use generative AI to generate any manuscript text, but help polish some sentences.

## CRediT authorship contribution statement

**Ling Yue:** Writing – review & editing, Writing – original draft, Resources, Funding acquisition, Data curation. **Yongsheng Pan:** Conceptualization. **Wei Li:** Writing – review & editing, Writing – original draft. **Junyan Mao:** Software, Data curation. **Bo Hong:** Writing – review & editing, Writing – original draft, Investigation, Data curation, Conceptualization. **Zhen Gu:** Writing – review & editing. **Mingxia Liu:** Writing – review & editing, Supervision, Investigation, Conceptualization. **Dinggang Shen:** Supervision, Investigation, Conceptualization. **Shifu Xiao:** Investigation, Funding acquisition, Conceptualization.

## Declaration of competing interest

The authors declare that they have no known competing financial interests or personal relationships that could have appeared to influence the work reported in this paper.

## Appendix Supplementary materials

Image, application 1

Image, application 2
