# Supplementary material for: Predicting cognitive decline: Deep-learning reveals subtle brain changes in pre-MCI stage
Source: J Prev Alzheimers Dis. 2025 Feb 6;12(5):100079. doi: 10.1016/j.tjpad.2025.100079 (PMC12183975; doi:10.1016/j.tjpad.2025.100079)
Supplement: Supplementary file 1 [file mmc1.docx]

**eTable 1 Demographics for the ADNI 1 dataset**

| **Demographics** | **Positive** | | | | **negative** | | |
| --- | --- | --- | --- | --- | --- | --- | --- |
|  | **AD** | **pNC** | **pMCI** | **MCI** | **sNC** | **sMCI** | **NC** |
| Number | 205 | 17 | 165 | 97 | 175 | 147 | 39 |
| male/female | 106/99 | 11/6 | 100/65 | 62/35 | 87/88 | 101/46 | 21/18 |
| Age(mean±sd) | 75.6±7.6 | 77.9±5.4 | 74.7±7.0 | 75.1±8.1 | 75.6±5.0 | 74.6±7.7 | 77.1±4.4 |

**Note:** p, progressive; s, stable
