# Supplementary material for: Predicting cognitive decline: Deep-learning reveals subtle brain changes in pre-MCI stage
Source: J Prev Alzheimers Dis. 2025 Feb 6;12(5):100079. doi: 10.1016/j.tjpad.2025.100079 (PMC12183975; doi:10.1016/j.tjpad.2025.100079)
Supplement: Supplementary file 2 [file mmc2.docx]

| **Demographics** | **Positive** | | **Negative** | | **Remaining** | |
| --- | --- | --- | --- | --- | --- | --- |
|  | **pNC** | **pSCD** | **sNC** | **sSCD** | **NC** | **SCD** |
| Number | 18 | 11 | 113 | 5 | 78 | 96 |
| male/female | 11/7 | 7/4 | 54/59 | 1/5 | 34/44 | 40/56 |
| age(mean±sd) | 77.50±6.75 | 74.82±6.93 | 72.78±5.83 | 72.60±6.27 | 72.72±6.71 | 71.97±5.26 |

**eTable 2 Demographics for the ADNI 2 dataset**

**Note:** p, progressive; s, stable
